# Supplementary material for: Proteotranscriptomic Profiling of the Toxic Mucus of Kulikovia alborostrata (Pilidiophora, Nemertea)
Source: Toxins (Basel). 2024 Dec 26;17(1):5. doi: 10.3390/toxins17010005 (PMC11768458; doi:10.3390/toxins17010005)
Supplement: Supplementary file 1 [file toxins-17-00005-s001.zip › Supplementary File S4.zip!/ORF 006024 LINALB/230688690e3e92c6/hit_summary.html]

Phyre 2 Results for ORF\_006024\_LINALB

|  |  |
| --- | --- |
| Email | vas9i-kz@mail.ru |
| Description | ORF\_006024\_LINALB |
| Date | Fri Apr 7 01:53:58 BST 2023 || Unique Job ID | 230688690e3e92c6 |

|  |  |
| --- | --- |
| Detailed template information |  |

| # | Template | Alignment Coverage | 3D Model | Confidence | % i.d. | Template Information |
| --- | --- | --- | --- | --- | --- | --- |

|  |  |  |  |  |  |  |
| --- | --- | --- | --- | --- | --- | --- |
| 1 | c6av8A\_ | --- \| --- \| --- |  | 88.4 | 48 | **PDB header:**toxin **Chain:** A: **PDB Molecule:**u5-theraphotoxin-hs1b 1;  **PDBTitle:** exploring cystine dense peptide space to open a unique molecular2 toolbox  **PDB Entry:** PDBe RCSB PDBj |
| 2 | c7rz3A\_ | --- \| --- \| --- |  | 38.5 | 58 | **PDB header:**toxin **Chain:** A: **PDB Molecule:**xt3a;  **PDBTitle:** the solution structure of remipede double-ick toxin phi-xibalbin3-xt3a  **PDB Entry:** PDBe RCSB PDBj |
| 3 | c5t4rA\_ | --- \| --- \| --- |  | 51.3 | 50 | **PDB header:**toxin **Chain:** A: **PDB Molecule:**mu-theraphotoxin-pn3a;  **PDBTitle:** nmr solution structure of the nav1.7 selective spider venom-derived2 peptide pn3a  **PDB Entry:** PDBe RCSB PDBj |
| 4 | c5we3A\_ | --- \| --- \| --- |  | 42.1 | 61 | **PDB header:**toxin **Chain:** A: **PDB Molecule:**beta-theraphotoxin-ps1a;  **PDBTitle:** solution nmr structure of paurtx-3  **PDB Entry:** PDBe RCSB PDBj |
| 5 | d1qk7a\_ | --- \| --- \| --- |  | 94.6 | 52 | **Fold:** Knottins (small inhibitors, toxins, lectins) **Superfamily:** omega toxin-like **Family:** Spider toxins **PDB entry:** PDBe RCSB PDBj |
| 6 | c6f61A\_ | --- \| --- \| --- |  | 50.5 | 42 | **PDB header:**antimicrobial protein **Chain:** A: **PDB Molecule:**purotoxin-6;  **PDBTitle:** nmr structure of purotoxin-6  **PDB Entry:** PDBe RCSB PDBj |
| 7 | c7skcA\_ | --- \| --- \| --- |  | 84.5 | 56 | **PDB header:**toxin **Chain:** A: **PDB Molecule:**ssp1a toxin;  **PDBTitle:** solution structure of spider toxin ssp1a  **PDB Entry:** PDBe RCSB PDBj |
| 8 | c1s6xA\_ | --- \| --- \| --- |  | 74.1 | 48 | **PDB header:**toxin **Chain:** A: **PDB Molecule:**kvap channel;  **PDBTitle:** solution structure of vstx  **PDB Entry:** PDBe RCSB PDBj |
| 9 | c1qfdA\_ | --- \| --- \| --- |  | 16.4 | 48 | **PDB header:**inhibitor **Chain:** A: **PDB Molecule:**protein (alpha-amylase inhibitor);  **PDBTitle:** nmr solution structure of alpha-amylase inhibitor (aai)  **PDB Entry:** PDBe RCSB PDBj |
| 10 | d1clvi\_ | --- \| --- \| --- |  | 13.6 | 48 | **Fold:** Knottins (small inhibitors, toxins, lectins) **Superfamily:** Plant inhibitors of proteinases and amylases **Family:** Plant inhibitors of proteinases and amylases **PDB entry:** PDBe RCSB PDBj |
| 11 | c8feyA\_ | --- \| --- \| --- |  | 77.7 | 53 | **PDB header:**toxin **Chain:** A: **PDB Molecule:**pmu1a toxin;  **PDBTitle:** solution structure of pmu1a  **PDB Entry:** PDBe RCSB PDBj |
| 12 | d1en2a2 | --- \| --- \| --- |  | 30.9 | 53 | **Fold:** Knottins (small inhibitors, toxins, lectins) **Superfamily:** Plant lectins/antimicrobial peptides **Family:** Hevein-like agglutinin (lectin) domain **PDB entry:** PDBe RCSB PDBj |
| 13 | d1ehda2 | --- \| --- \| --- |  | 29.3 | 53 | **Fold:** Knottins (small inhibitors, toxins, lectins) **Superfamily:** Plant lectins/antimicrobial peptides **Family:** Hevein-like agglutinin (lectin) domain **PDB entry:** PDBe RCSB PDBj |
| 14 | c7namB\_ | --- \| --- \| --- |  | 73.8 | 59 | **PDB header:**signaling protein **Chain:** B: **PDB Molecule:**trypsin inhibitor 2;  **PDBTitle:** lrp6\_e1 in complex with lr-eet-3.5  **PDB Entry:** PDBe RCSB PDBj |
| 15 | c6f8eA\_ | --- \| --- \| --- |  | 12.7 | 59 | **PDB header:**lipid binding protein **Chain:** A: **PDB Molecule:**pleckstrin homology domain;  **PDBTitle:** ph domain from tgaph  **PDB Entry:** PDBe RCSB PDBj |
| 16 | c2n86A\_ | --- \| --- \| --- |  | 15.3 | 40 | **PDB header:**toxin **Chain:** A: **PDB Molecule:**spiderine-1a;  **PDBTitle:** nmr structure of ottx1a - ick  **PDB Entry:** PDBe RCSB PDBj |
| 17 | c1zwuA\_ | --- \| --- \| --- |  | 9.4 | 43 | **PDB header:**antimicrobial protein **Chain:** A: **PDB Molecule:**amaranthus caudatus antimicrobial peptide 2 (acmp2);  **PDBTitle:** 30 nmr structures of acamp2-like peptide with non natural beta-(2-2 naphthyl)-alanine residue.  **PDB Entry:** PDBe RCSB PDBj |
| 18 | c2m9lA\_ | --- \| --- \| --- |  | 57.4 | 41 | **PDB header:**toxin **Chain:** A: **PDB Molecule:**beta-theraphotoxin-tp1a;  **PDBTitle:** solution structure of protoxin-1  **PDB Entry:** PDBe RCSB PDBj |
| 19 | c2a2vA\_ | --- \| --- \| --- |  | 23.0 | 41 | **PDB header:**toxin **Chain:** A: **PDB Molecule:**jingzhaotoxin-xi;  **PDBTitle:** the solution structure of jingzhaotoxin-xi  **PDB Entry:** PDBe RCSB PDBj |
| 20 | c2jtbA\_ | --- \| --- \| --- |  | 77.1 | 60 | **PDB header:**toxin **Chain:** A: **PDB Molecule:**hainantoxin-3;  **PDBTitle:** three dimensional solution structure of hainantoxin-iii by 2d 1h-nmr  **PDB Entry:** PDBe RCSB PDBj |
| 21 | c1qk6A\_ | --- \| --- \| --- | not modelled | 58.7 | 50 | **PDB header:**toxin **Chain:** A: **PDB Molecule:**huwentoxin-i;  **PDBTitle:** solution structure of huwentoxin-i by nmr  **PDB Entry:** PDBe RCSB PDBj |
| 22 | c7jpmA\_ | --- \| --- \| --- | not modelled | 21.7 | 50 | **PDB header:**toxin **Chain:** A: **PDB Molecule:**omega-theraphotoxin-pm1b;  **PDBTitle:** the solution structure of omega-theraphotoxin-pm1b isolated from king2 baboon spider  **PDB Entry:** PDBe RCSB PDBj |
| 23 | c6mk5A\_ | --- \| --- \| --- | not modelled | 21.3 | 50 | **PDB header:**toxin **Chain:** A: **PDB Molecule:**toxin gtx1-15;  **PDBTitle:** solution nmr structure of spider toxin analogue [f5a,m6f,t26l,2 k28r]gptx-1  **PDB Entry:** PDBe RCSB PDBj |
| 24 | d1tyka\_ | --- \| --- \| --- | not modelled | 9.6 | 50 | **Fold:** Knottins (small inhibitors, toxins, lectins) **Superfamily:** omega toxin-like **Family:** Spider toxins **PDB entry:** PDBe RCSB PDBj |
| 25 | c2wh9A\_ | --- \| --- \| --- | not modelled | 90.0 | 64 | **PDB header:**membrane protein inhibitor **Chain:** A: **PDB Molecule:**guangxitoxin-1egxtx-1e;  **PDBTitle:** solution structure of gxtx-1e  **PDB Entry:** PDBe RCSB PDBj |
| 26 | d1eita\_ | --- \| --- \| --- | not modelled | 34.6 | 56 | **Fold:** Knottins (small inhibitors, toxins, lectins) **Superfamily:** omega toxin-like **Family:** Spider toxins **PDB entry:** PDBe RCSB PDBj |
| 27 | c1eitA\_ | --- \| --- \| --- | not modelled | 34.6 | 56 | **PDB header:**neurotoxin **Chain:** A: **PDB Molecule:**mu-agatoxin-i;  **PDBTitle:** nmr study of mu-agatoxin  **PDB Entry:** PDBe RCSB PDBj |
| 28 | c2aapA\_ | --- \| --- \| --- | not modelled | 40.6 | 47 | **PDB header:**toxin **Chain:** A: **PDB Molecule:**jingzhaotoxin-vii;  **PDBTitle:** solution structure of jingzhaotoxin-vii  **PDB Entry:** PDBe RCSB PDBj |
| 29 | c1emxA\_ | --- \| --- \| --- | not modelled | 67.9 | 62 | **PDB header:**toxin **Chain:** A: **PDB Molecule:**heteropodatoxin 2;  **PDBTitle:** solution structure of hptx2, a toxin from heteropoda venatoria spider2 venom that blocks kv4.2 potassium channel  **PDB Entry:** PDBe RCSB PDBj |
| 30 | c6ceiA\_ | --- \| --- \| --- | not modelled | 75.0 | 50 | **PDB header:**toxin **Chain:** A: **PDB Molecule:**gxia;  **PDBTitle:** solution nmr structure of conotoxin gxia from conus geographus  **PDB Entry:** PDBe RCSB PDBj |
| 31 | c2n3pA\_ | --- \| --- \| --- | not modelled | 13.5 | 32 | **PDB header:**toxin **Chain:** A: **PDB Molecule:**asteropsin\_g;  **PDBTitle:** solution nmr structure of asteropsin g from marine sponge asteropus  **PDB Entry:** PDBe RCSB PDBj |
| 32 | c5yjhA\_ | --- \| --- \| --- | not modelled | 25.5 | 47 | **PDB header:**cell adhesion **Chain:** A: **PDB Molecule:**periostin;  **PDBTitle:** structural insights into periostin functions  **PDB Entry:** PDBe RCSB PDBj |
| 33 | c6f24A\_ | --- \| --- \| --- | not modelled | 11.7 | 47 | **PDB header:**lipid binding protein **Chain:** A: **PDB Molecule:**c-terminal ph domain from pfaph;  **PDBTitle:** ph domain from pfaph  **PDB Entry:** PDBe RCSB PDBj |
| 34 | c5nv6A\_ | --- \| --- \| --- | not modelled | 10.0 | 47 | **PDB header:**structural protein **Chain:** A: **PDB Molecule:**transforming growth factor-beta-induced protein ig-h3;  **PDBTitle:** structure of human transforming growth factor beta-induced protein2 (tgfbip).  **PDB Entry:** PDBe RCSB PDBj |
| 35 | c1kozA\_ | --- \| --- \| --- | not modelled | 44.7 | 42 | **PDB header:**toxin **Chain:** A: **PDB Molecule:**voltage-dependent channel inhibitor;  **PDBTitle:** solution structure of omega-grammotoxin sia  **PDB Entry:** PDBe RCSB PDBj |
| 36 | c1mcvI\_ | --- \| --- \| --- | not modelled | 22.2 | 57 | **PDB header:**hydrolase **Chain:** I: **PDB Molecule:**hei-toe i;  **PDBTitle:** crystal structure analysis of a hybrid squash inhibitor in2 complex with porcine pancreatic elastase  **PDB Entry:** PDBe RCSB PDBj |
| 37 | d1mcvi\_ | --- \| --- \| --- | not modelled | 22.2 | 57 | **Fold:** Knottins (small inhibitors, toxins, lectins) **Superfamily:** Plant inhibitors of proteinases and amylases **Family:** Plant inhibitors of proteinases and amylases **PDB entry:** PDBe RCSB PDBj |
| 38 | d2it7a1 | --- \| --- \| --- | not modelled | 21.9 | 57 | **Fold:** Knottins (small inhibitors, toxins, lectins) **Superfamily:** Plant inhibitors of proteinases and amylases **Family:** Plant inhibitors of proteinases and amylases **PDB entry:** PDBe RCSB PDBj |
| 39 | c2it7A\_ | --- \| --- \| --- | not modelled | 21.9 | 57 | **PDB header:**plant protein **Chain:** A: **PDB Molecule:**trypsin inhibitor 2;  **PDBTitle:** solution structure of the squash trypsin inhibitor eeti-ii  **PDB Entry:** PDBe RCSB PDBj |
| 40 | c2letA\_ | --- \| --- \| --- | not modelled | 21.7 | 57 | **PDB header:**proteinase inhibitor(trypsin) **Chain:** A: **PDB Molecule:**trypsin inhibitor ii;  **PDBTitle:** an 1h nmr determination of the three dimensional structures of mirror2 image forms of a leu-5 variant of the trypsin inhibitor ecballium3 elaterium (eeti-ii)  **PDB Entry:** PDBe RCSB PDBj |
| 41 | d1h9hi\_ | --- \| --- \| --- | not modelled | 20.3 | 57 | **Fold:** Knottins (small inhibitors, toxins, lectins) **Superfamily:** Plant inhibitors of proteinases and amylases **Family:** Plant inhibitors of proteinases and amylases **PDB entry:** PDBe RCSB PDBj |
| 42 | c6a48A\_ | --- \| --- \| --- | not modelled | 13.9 | 42 | **PDB header:**signaling protein **Chain:** A: **PDB Molecule:**reelin;  **PDBTitle:** crystal structure of reelin n-terminal region  **PDB Entry:** PDBe RCSB PDBj |
| 43 | c6gftA\_ | --- \| --- \| --- | not modelled | 26.3 | 53 | **PDB header:**toxin **Chain:** A: **PDB Molecule:**cyriotoxin-1a;  **PDBTitle:** antinociceptive evaluation of cyriotoxin-1a, the first toxin purified2 from cyriopagopus schioedtei spider venom  **PDB Entry:** PDBe RCSB PDBj |
| 44 | d1h9ii\_ | --- \| --- \| --- | not modelled | 21.6 | 53 | **Fold:** Knottins (small inhibitors, toxins, lectins) **Superfamily:** Plant inhibitors of proteinases and amylases **Family:** Plant inhibitors of proteinases and amylases **PDB entry:** PDBe RCSB PDBj |
| 45 | c5i1xA\_ | --- \| --- \| --- | not modelled | 59.8 | 44 | **PDB header:**toxin **Chain:** A: **PDB Molecule:**mu-trtx-pre1a toxin;  **PDBTitle:** resonance assignments and nmr structure determination of tarantula2 toxin, f8a mutant of beta-trtx-pre1a  **PDB Entry:** PDBe RCSB PDBj |
| 46 | c2mt7A\_ | --- \| --- \| --- | not modelled | 56.3 | 44 | **PDB header:**toxin **Chain:** A: **PDB Molecule:**hs1a;  **PDBTitle:** solution structure of spider-venom peptide hs1a  **PDB Entry:** PDBe RCSB PDBj |
| 47 | c5o57A\_ | --- \| --- \| --- | not modelled | 21.1 | 44 | **PDB header:**signaling protein **Chain:** A: **PDB Molecule:**dickkopf-related protein 4;  **PDBTitle:** solution structure of the n-terminal region of dkk4  **PDB Entry:** PDBe RCSB PDBj |
| 48 | d1d1ha\_ | --- \| --- \| --- | not modelled | 16.2 | 36 | **Fold:** Knottins (small inhibitors, toxins, lectins) **Superfamily:** omega toxin-like **Family:** Spider toxins **PDB entry:** PDBe RCSB PDBj |
| 49 | d1la4a\_ | --- \| --- \| --- | not modelled | 16.1 | 36 | **Fold:** Knottins (small inhibitors, toxins, lectins) **Superfamily:** omega toxin-like **Family:** Spider toxins **PDB entry:** PDBe RCSB PDBj |
| 50 | c2kusA\_ | --- \| --- \| --- | not modelled | 12.2 | 36 | **PDB header:**antimicrobial protein **Chain:** A: **PDB Molecule:**sm-amp-1.1a;  **PDBTitle:** spatial structure of antimicrobial peptide sm-amp-1.1a  **PDB Entry:** PDBe RCSB PDBj |
| 51 | c2mvaA\_ | --- \| --- \| --- | not modelled | 11.2 | 44 | **PDB header:**toxin **Chain:** A: **PDB Molecule:**rhtx toxin;  **PDBTitle:** solution structure of the toxin, rhtx  **PDB Entry:** PDBe RCSB PDBj |
| 52 | d1nixa\_ | --- \| --- \| --- | not modelled | 20.7 | 47 | **Fold:** Knottins (small inhibitors, toxins, lectins) **Superfamily:** omega toxin-like **Family:** Spider toxins **PDB entry:** PDBe RCSB PDBj |
| 53 | c6nk9A\_ | --- \| --- \| --- | not modelled | 15.6 | 47 | **PDB header:**toxin **Chain:** A: **PDB Molecule:**aca toxin 1;  **PDBTitle:** solution structure of acatx1, a potassium channel inhibitor from the2 sea anemone antopleura cascaia  **PDB Entry:** PDBe RCSB PDBj |
| 54 | c2jyeA\_ | --- \| --- \| --- | not modelled | 20.5 | 32 | **PDB header:**cytokine **Chain:** A: **PDB Molecule:**granulin a;  **PDBTitle:** human granulin a  **PDB Entry:** PDBe RCSB PDBj |
| 55 | c6v6tA\_ | --- \| --- \| --- | not modelled | 14.1 | 32 | **PDB header:**toxin **Chain:** A: **PDB Molecule:**delta-theraphotoxin-hm1b;  **PDBTitle:** solution structure of delta-theraphotoxin-hm1b from heteroscodra2 maculata  **PDB Entry:** PDBe RCSB PDBj |
| 56 | c6btvA\_ | --- \| --- \| --- | not modelled | 69.8 | 39 | **PDB header:**toxin **Chain:** A: **PDB Molecule:**beta-theraphotoxin-cm1b;  **PDBTitle:** solution nmr structures for ccotx-ii  **PDB Entry:** PDBe RCSB PDBj |
| 57 | c5epmC\_ | --- \| --- \| --- | not modelled | 41.7 | 39 | **PDB header:**toxin/immune system **Chain:** C: **PDB Molecule:**beta-theraphotoxin-cm1a;  **PDBTitle:** ceratotoxin variant in complex with specific antibody fab fragment  **PDB Entry:** PDBe RCSB PDBj |
| 58 | c2i1tA\_ | --- \| --- \| --- | not modelled | 37.9 | 50 | **PDB header:**toxin **Chain:** A: **PDB Molecule:**jingzhaotoxin-3;  **PDBTitle:** solution structure of jingzhaotoxin-iii, a novel toxin inhibiting both2 nav and kv channels  **PDB Entry:** PDBe RCSB PDBj |
| 59 | c5ki9A\_ | --- \| --- \| --- | not modelled | 31.1 | 70 | **PDB header:**antimicrobial protein **Chain:** A: **PDB Molecule:**beta-defensin 104;  **PDBTitle:** crystal structure of human beta-defensin 4 (hbd4)  **PDB Entry:** PDBe RCSB PDBj |
| 60 | c6lnrA\_ | --- \| --- \| --- | not modelled | 15.4 | 50 | **PDB header:**hydrolase **Chain:** A: **PDB Molecule:**class-1 chitinase;  **PDBTitle:** structure of intact chitinase with hevein domain from the plant2 simarouba glauca, known for its traditional anti-inflammatory3 efficacy  **PDB Entry:** PDBe RCSB PDBj |
| 61 | c2ys0A\_ | --- \| --- \| --- | not modelled | 10.3 | 50 | **PDB header:**hydrolase **Chain:** A: **PDB Molecule:**ectonucleotide pyrophosphatase/phosphodiesterase family  **PDBTitle:** solution structure of the somatomedin b domain of human ectonucleotide2 pyrophosphatase/phosphodiesterase family member  **PDB Entry:** PDBe RCSB PDBj |
| 62 | c6ji7A\_ | --- \| --- \| --- | not modelled | 55.8 | 58 | **PDB header:**plant protein **Chain:** A: **PDB Molecule:**coffeetide;  **PDBTitle:** coffeetides: iron-binding cysteine rich peptides from coffee waste  **PDB Entry:** PDBe RCSB PDBj |
| 63 | c1omgA\_ | --- \| --- \| --- | not modelled | 15.3 | 58 | **PDB header:**presynaptic neurotoxin **Chain:** A: **PDB Molecule:**omega-conotoxin mviia;  **PDBTitle:** nmr study of omega-conotoxin mviia  **PDB Entry:** PDBe RCSB PDBj |
| 64 | c1dw4A\_ | --- \| --- \| --- | not modelled | 15.3 | 58 | **PDB header:**toxin **Chain:** A: **PDB Molecule:**omega-conotoxin mviia;  **PDBTitle:** nmr structure of omega-conotoxin mviia: constraints on disulphide2 bridges  **PDB Entry:** PDBe RCSB PDBj |
| 65 | c7mixB\_ | --- \| --- \| --- | not modelled | 15.3 | 58 | **PDB header:**transport protein/toxin **Chain:** B: **PDB Molecule:**omega-conotoxin mviia;  **PDBTitle:** human n-type voltage-gated calcium channel cav2.2 in the presence of2 ziconotide at 3.0 angstrom resolution  **PDB Entry:** PDBe RCSB PDBj |
| 66 | c1tt3A\_ | --- \| --- \| --- | not modelled | 15.2 | 58 | **PDB header:**toxin **Chain:** A: **PDB Molecule:**omega-conotoxin mviia;  **PDBTitle:** nmr soulution structure of omega-conotoxin [k10]mviia  **PDB Entry:** PDBe RCSB PDBj |
| 67 | c1mviA\_ | --- \| --- \| --- | not modelled | 15.1 | 58 | **PDB header:**neurotoxin **Chain:** A: **PDB Molecule:**mviia;  **PDBTitle:** n-type calcium channel blocker, omega-conotoxin mviia, nmr, 152 structures  **PDB Entry:** PDBe RCSB PDBj |
| 68 | c1dw5A\_ | --- \| --- \| --- | not modelled | 15.1 | 58 | **PDB header:**toxin **Chain:** A: **PDB Molecule:**omega-conotoxin mviia;  **PDBTitle:** nmr structure of omega-conotoxin mviia: no constraints on disulphide2 bridges  **PDB Entry:** PDBe RCSB PDBj |
| 69 | c7vfuE\_ | --- \| --- \| --- | not modelled | 15.0 | 58 | **PDB header:**membrane protein **Chain:** E: **PDB Molecule:**omega-conotoxin mviia;  **PDBTitle:** human n-type voltage gated calcium channel cav2.2-alpha2/delta1-beta12 complex, bound to ziconotide  **PDB Entry:** PDBe RCSB PDBj |
| 70 | c1ttkA\_ | --- \| --- \| --- | not modelled | 14.9 | 58 | **PDB header:**toxin **Chain:** A: **PDB Molecule:**omega-conotoxin mviia;  **PDBTitle:** nmr solution structure of omega-conotoxin mviia, a n-type calcium2 channel blocker  **PDB Entry:** PDBe RCSB PDBj |
| 71 | c1feoA\_ | --- \| --- \| --- | not modelled | 14.1 | 58 | **PDB header:**toxin **Chain:** A: **PDB Molecule:**omega-conotoxin mviia-gly;  **PDBTitle:** solution structure of omega-conotoxin mviia with c-terminal gly  **PDB Entry:** PDBe RCSB PDBj |
| 72 | c3s8vX\_ | --- \| --- \| --- | not modelled | 11.8 | 38 | **PDB header:**signaling protein **Chain:** X: **PDB Molecule:**dickkopf-related protein 1;  **PDBTitle:** crystal structure of lrp6-dkk1 complex  **PDB Entry:** PDBe RCSB PDBj |
| 73 | d1hp3a\_ | --- \| --- \| --- | not modelled | 10.6 | 55 | **Fold:** Knottins (small inhibitors, toxins, lectins) **Superfamily:** omega toxin-like **Family:** Spider toxins **PDB entry:** PDBe RCSB PDBj |
| 74 | c1hp3A\_ | --- \| --- \| --- | not modelled | 10.6 | 55 | **PDB header:**toxin **Chain:** A: **PDB Molecule:**omega-atracotoxin-hv2a;  **PDBTitle:** c-terminal truncation of omega-atracotoxin-hv2a (ct-hv2a)  **PDB Entry:** PDBe RCSB PDBj |
| 75 | c2dcwA\_ | --- \| --- \| --- | not modelled | 9.9 | 55 | **PDB header:**antimicrobial protein **Chain:** A: **PDB Molecule:**tachystatin-b2;  **PDBTitle:** the solution structure of horseshoe crab antimicrobial peptide2 tachystatin b with the inhibitory cystine-knot motif  **PDB Entry:** PDBe RCSB PDBj |
| 76 | c2dcvA\_ | --- \| --- \| --- | not modelled | 9.8 | 55 | **PDB header:**antimicrobial protein **Chain:** A: **PDB Molecule:**tachystatin-b1;  **PDBTitle:** the solution structure of horseshoe crab antimicrobial peptide2 tachystatin b with the inhibitory cystine-knot motif  **PDB Entry:** PDBe RCSB PDBj |
| 77 | c1eyoA\_ | --- \| --- \| --- | not modelled | 10.7 | 67 | **PDB header:**toxin **Chain:** A: **PDB Molecule:**conotoxin tviia;  **PDBTitle:** solution structure of conotoxin tviia from conus tulipa  **PDB Entry:** PDBe RCSB PDBj |
| 78 | c1v91A\_ | --- \| --- \| --- | not modelled | 13.4 | 75 | **PDB header:**toxin **Chain:** A: **PDB Molecule:**delta-palutoxin it2;  **PDBTitle:** solution structure of insectidal toxin delta-paluit2-nh2  **PDB Entry:** PDBe RCSB PDBj |
| 79 | c6xyiA\_ | --- \| --- \| --- | not modelled | 10.0 | 60 | **PDB header:**toxin **Chain:** A: **PDB Molecule:**ams9.3.1;  **PDBTitle:** nmr solution structure of alpha-anmtx- ms11a-3 (ms11a-3)  **PDB Entry:** PDBe RCSB PDBj |
| 80 | c6xyhA\_ | --- \| --- \| --- | not modelled | 9.9 | 60 | **PDB header:**toxin **Chain:** A: **PDB Molecule:**ams3;  **PDBTitle:** nmr solution structure of alpha-anmtx-ms11a-2 (ms11a-2)  **PDB Entry:** PDBe RCSB PDBj |
| 81 | d1e4ra\_ | --- \| --- \| --- | not modelled | 9.6 | 50 | **Fold:** Defensin-like **Superfamily:** Defensin-like **Family:** Defensin **PDB entry:** PDBe RCSB PDBj |
| 82 | c7lyuA\_ | --- \| --- \| --- | not modelled | 15.9 | 35 | **PDB header:**signaling protein **Chain:** A: **PDB Molecule:**reelin;  **PDBTitle:** reelin repeat 8  **PDB Entry:** PDBe RCSB PDBj |
| 83 | c2kraA\_ | --- \| --- \| --- | not modelled | 11.9 | 35 | **PDB header:**contractile protein **Chain:** A: **PDB Molecule:**prokineticin bv8;  **PDBTitle:** solution structure of bv8  **PDB Entry:** PDBe RCSB PDBj |
| 84 | c1imtA\_ | --- \| --- \| --- | not modelled | 10.9 | 35 | **PDB header:**toxin **Chain:** A: **PDB Molecule:**intestinal toxin 1;  **PDBTitle:** mamba intestinal toxin 1, nmr, 39 structures  **PDB Entry:** PDBe RCSB PDBj |
| 85 | d1ayja\_ | --- \| --- \| --- | not modelled | 12.8 | 56 | **Fold:** Knottins (small inhibitors, toxins, lectins) **Superfamily:** Scorpion toxin-like **Family:** Plant defensins **PDB entry:** PDBe RCSB PDBj |
| 86 | c1fu3A\_ | --- \| --- \| --- | not modelled | 9.7 | 63 | **PDB header:**metal transport inhibitor **Chain:** A: **PDB Molecule:**delta-conotoxin txvia;  **PDBTitle:** three-dimensional structure in solution of the sodium channel2 agonist/antagonist delta-conotoxin txvia  **PDB Entry:** PDBe RCSB PDBj |
| 87 | d1fu3a\_ | --- \| --- \| --- | not modelled | 9.7 | 63 | **Fold:** Knottins (small inhibitors, toxins, lectins) **Superfamily:** omega toxin-like **Family:** Conotoxin **PDB entry:** PDBe RCSB PDBj |
| 88 | c5znuA\_ | --- \| --- \| --- | not modelled | 16.8 | 83 | **PDB header:**toxin **Chain:** A: **PDB Molecule:**omega-conotoxin-like bu8;  **PDBTitle:** structure of omega conotoxin bu8  **PDB Entry:** PDBe RCSB PDBj |
| 89 | c1mvjA\_ | --- \| --- \| --- | not modelled | 16.2 | 83 | **PDB header:**neurotoxin **Chain:** A: **PDB Molecule:**svib;  **PDBTitle:** n-type calcium channel blocker, omega-conotoxin mviia nmr, 152 structures  **PDB Entry:** PDBe RCSB PDBj |
| 90 | c1cnnA\_ | --- \| --- \| --- | not modelled | 16.0 | 83 | **PDB header:**toxin **Chain:** A: **PDB Molecule:**omega-conotoxin mviic;  **PDBTitle:** omega-conotoxin mviic from conus magus  **PDB Entry:** PDBe RCSB PDBj |
| 91 | c1omnA\_ | --- \| --- \| --- | not modelled | 15.7 | 83 | **PDB header:**neurotoxin **Chain:** A: **PDB Molecule:**omega-conotoxin m vii c (m seven c);  **PDBTitle:** solution structure of omega-conotoxin mviic, a high affinity of p-type2 calcium channels, using 1h nmr spectroscopy and complete relaxation3 matrix analysis  **PDB Entry:** PDBe RCSB PDBj |
| 92 | c3q8jA\_ | --- \| --- \| --- | not modelled | 14.5 | 83 | **PDB header:**toxin **Chain:** A: **PDB Molecule:**asteropsin a;  **PDBTitle:** crystal structure of asteropsin a from marine sponge asteropus sp.  **PDB Entry:** PDBe RCSB PDBj |
| 93 | c1v4qA\_ | --- \| --- \| --- | not modelled | 13.4 | 83 | **PDB header:**toxin **Chain:** A: **PDB Molecule:**omega-conotoxin mviic;  **PDBTitle:** three-dimensional solution structure of the analogue peptide of omega-2 conotoxin mviic  **PDB Entry:** PDBe RCSB PDBj |
| 94 | c2n7fA\_ | --- \| --- \| --- | not modelled | 20.6 | 71 | **PDB header:**toxin **Chain:** A: **PDB Molecule:**muo-conotoxin mfvia;  **PDBTitle:** nmr solution structure of muo-conotoxin mfvia  **PDB Entry:** PDBe RCSB PDBj |
| 95 | c1rmkA\_ | --- \| --- \| --- | not modelled | 19.7 | 71 | **PDB header:**toxin **Chain:** A: **PDB Molecule:**mu-o-conotoxin mrvib;  **PDBTitle:** solution structure of conotoxin mrvib  **PDB Entry:** PDBe RCSB PDBj |
| 96 | d1rmka\_ | --- \| --- \| --- | not modelled | 19.7 | 71 | **Fold:** Knottins (small inhibitors, toxins, lectins) **Superfamily:** omega toxin-like **Family:** Conotoxin **PDB entry:** PDBe RCSB PDBj |
| 97 | d3c8ya1 | --- \| --- \| --- | not modelled | 16.1 | 71 | **Fold:** Fe-only hydrogenase **Superfamily:** Fe-only hydrogenase **Family:** Fe-only hydrogenase **PDB entry:** PDBe RCSB PDBj |
| 98 | c3lx4B\_ | --- \| --- \| --- | not modelled | 19.3 | 57 | **PDB header:**oxidoreductase **Chain:** B: **PDB Molecule:**fe-hydrogenase;  **PDBTitle:** stepwise [fefe]-hydrogenase h-cluster assembly revealed in the2 structure of hyda(deltaefg)  **PDB Entry:** PDBe RCSB PDBj |
| 99 | d1hfel1 | --- \| --- \| --- | not modelled | 11.0 | 57 | **Fold:** Fe-only hydrogenase **Superfamily:** Fe-only hydrogenase **Family:** Fe-only hydrogenase **PDB entry:** PDBe RCSB PDBj |
